# Whole-genome sequence of *Salmonella* Typhimurium E-5591 and its methionine sulfoxide reductase (*msr*) gene deletion mutant strains

**DOI:** 10.1128/mra.00143-25

**Published:** 2025-06-10

**Authors:** Esha Sinha, Tapan Kumar Singh Chauhan, Suchitra Upreti, Raj Sahoo, Arijit Shome, Lalhmangaihzuali Lalhmangaihzuali, Shikha Bishnoi, Mukesh Kumar, Irungbam Karuna Devi, Prasad Thomas, Mithilesh Kumar Singh, Salauddin Qureshi, Manish Mahawar

**Affiliations:** ^1^Divisions of Biological Standardization, ICAR-Indian Veterinary Research Institute, Bareilly, India; 2 Bacteriology and Mycology, ICAR-Indian Veterinary Research Institute, Bareilly, India; 3 Biochemistry, ICAR-Indian Veterinary Research Institute, Bareilly, India; 4 Immunology-section, ICAR-Indian Veterinary Research Institute, Bareilly, India; University of Maryland School of Medicine, Baltimore, Maryland, USA

**Keywords:** *Salmonella*, oxidative stress, methionine sulfoxide reductase

## Abstract

*Salmonella* Typhimurium is one of the leading causes of foodborne illness in humans. Poultry products are the main source of infection for humans. Here we report the whole-genome sequences of *Salmonella* Typhimurium strain E-5591 (a field poultry isolate) and its mutant strains lacking the various methionine sulfoxide reductase genes.

## ANNOUNCEMENT

*Salmonella* enterica serovar Typhimurium is a non-typhoidal serovar of *Salmonella* which is associated with foodborne gastroenteritis in humans. Poultry birds serve as the asymptomatic reservoir for this bacterium. The transmission to humans occurs mainly through the consumption of contaminated poultry products, highlighting its zoonotic importance and risks to the food chain (1). *Salmonella* Typhimurium strain E-5591 isolated from poultry muscle tissue was procured from the repository of the National *Salmonella* Centre (Veterinary Type), ICAR-Indian Veterinary Research Institute, Izatnagar, India. The E-5591 strain of *Salmonella* Typhimurium showed more efficient cecal colonization in poultry than other tested strains (E-2375, E-4231, E-4831, and E-5587) (2).

*Salmonella* is a facultative intracellular bacterium capable of surviving and replicating in the oxidative environment of macrophages and neutrophils. These phagocytic cells generate reactive oxygen species, such as hydrogen peroxide and hydroxyl radicals, that oxidize free or protein-bound methionine residues to methionine sulfoxides (Met-SO), compromising cellular survival. Methionine sulfoxide reductases (*msr*’s) repair Met-SO and enhance the survival of *Salmonella* in the oxidative environment of macrophages (3). *Salmonella* Typhimurium encodes five *msr*s: *msrA*, *msrB*, *msrC* (4), *msrP* (5), and *bisC* (6). Earlier, we generated various *msr* gene deletion strains in *Salmonella* Typhimurium (7–11). The deletions were confirmed by PCR.

The freezer stocks of *Salmonella* Typhimurium and various mutant strains (Δ*msrA*, Δ*msrP*, Δ*msrAC*, Δ*msrACP*, Δ*msrACPB*, and Δ*msrACPBbisC*) were streaked on Hektoen enteric agar and incubated at 37°C for 16 hours. Next day, isolated colonies were inoculated and grown in 10 mL of Luria-Bertani broth at 37°C for 14–16 hours. The cultures were pelleted to isolate genomic DNA. The genomic DNA from the mutant strains was isolated by the PureLink Genomic DNA Mini Kit (cat. no. K182002, Invitrogen), according to the manufacturer’s protocol. The purity of the extracted DNA was assessed using a NanoDrop spectrophotometer (Thermo Fisher Scientific, Waltham, MA). We used Twist Library Preparation Kit v.2.0 (cat. no. 104206, Twist Bioscience) with Enzymatic Fragmentation and Twist Universal Adapter System for library preparation. The whole-genome sequencing was performed using the Illumina NovaSeq 6000 platform (N2Jenomics Lab Pvt. Ltd., New Delhi, India). Paired-end libraries with 150 bp reads were generated. Sequence adaptors and low-quality reads were filtered using Adapter Removal v.2.3.2. (https://github.com/MikkelSchubert/adapterremoval) (12). Phred quality score threshold was set at 30. The quality filtered data were aligned to the reference genome using NC_003197.2 and NC_003277.2 sequences using bowtie2 v.2.4.4 (https://github.com/BenLangmead/bowtie2/releases) (13). The variant calling was performed based on the reference genome using bcftools v.1.13 (https://github.com/samtools/bcftools/releases/) (14). The *de novo* assembly was done using Unicycler v.0.4.8 (https://github.com/rrwick/Unicycler) (15). The downstream annotation was performed using the prokaryotic gene-prediction tool Prokka v.1.14.6 (https://github.com/tseemann/prokka) (16). The assembly statistics were generated using QUAST v.5.0.2 (https://quast.sourceforge.net/download.html) (17). The cassette sequences were identified in the assembly based on the homology search using blastn (BLAST v.2.12.0+) (https://ncbiinsights.ncbi.nlm.nih.gov/2021/07/09/blast-2-12-0/) (18) with the cassette sequences used to delete the targeted gene. Default parameters were used for all bioinformatics tools. The genomic features of *Salmonella* Typhimurium and its *msr* mutant strains are presented in Table 1.

**TABLE 1:** Genomic features of *Salmonella* Typhimurium and *msr* mutant strains: assembly statistics and sequence data

Name of strain	No. of contigs	Assembly size (Mbp)	*N*_50_ (bp)	No. of reads	GC%	SRA^*a*^ accession no.
*Salmonella* Typhimurium E-5591	46	4.70	353,339	13,589,737	52.12	SRX25641495
Δ*msrA*	61	4.96	225,558	16,477,527	52.16	SRX25462152
Δ*msrP*	44	4.70	356,425	4,600,526	52.12	SRX25657319
Δ*msrAC*	61	4.96	225,558	17,168,347	52.16	SRX25649665
Δ*msrACP*	61	4.96	225,558	17,667,128	52.16	SRX25657042
Δ*msrACPB*	60	4.96	271,146	3,603,428	52.16	SRX23226708
Δ*msrACPBBisC*	59	4.96	271,146	3,684,657	52.16	SRX23226707

^*a*^ SRA, Sequence Read Archive.

## Data Availability

The whole-genome sequencing data have been submitted to the National Center for Biotechnology Information under BioProject entitled "Methionine Sulfoxide Reductase (msr) Gene Deletion Strains of *Salmonella* Typhimurium in Poultry" with the BioProject ID PRJNA1064004. This BioProject includes seven biosamples, and the Illumina sequencing reads are available in the Sequence Read Archive under the accession numbers SRX25641495, SRX25462152, SRX25657319, SRX25649665, SRX25657042, SRX23226708, and SRX23226707.
